# Experimental credit

**DOI:** 10.1038/s44319-024-00105-w

**Published:** 2024-03-12

**Authors:** Bernd Pulverer

**Affiliations:** EMBO, Heidelberg, Germany

**Keywords:** Science Policy & Publishing

## Abstract

*EMBO Reports* asks authors to post the source data of every key experiment. Figure panel authorship allows scientist to sign their experiments for transparency, credit and accountability.

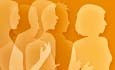

## Teaser

*EMBO Reports* asks authors to post source data of every key experiment. A free platform and curation support ensures this data archiving service does not become a chore. Figure panel authorship allows scientists to claim their experiments for transparency, credit and accountability.

The heart and soul of research is data, archived and described in a way that renders it interpretable and reproducible. In an Open Science world, that data should also be as FAIR (Findable, Accessible, Interoperable and Reusable; https://www.go-fair.org/fair-principles/) as possible. The interpretation and the “packaging” of research findings into human-readable “research stories” in papers or scientific talks, or, for a broader reach, as a review or for teaching, or indeed for social- and other media, are undeniably an essential skillset of any scientist who needs to succeed in a highly competitive research environment. Yet, without rock-solid data as the basis, the interpretation and framing will ultimately tumble like a house of cards, as per Efraim Racker’s quip “do not waste clean thinking on dirty enzymes”.

## The paper

And yet, decades into the digitization of the publishing process, we are still quasi-religiously focused on the classical research paper as the only platform to distribute scientific knowledge: text describing results, outlining methods, and offering conclusions and background, all supported by what are effectively visual illustrations of data in the form of figures. To be fair, database deposition has been a standard for years in many domains, but the beady eye of research assessment retains its tunnel vision on the paper alone. It of course makes sense that in a world aflush with more or less robust and more or less interesting data, the human brain needs to focus on the bare essentials. Provision of this stratification is the domain of the peer-reviewed, selective scientific journal. This has remained true even with the advent of advanced search engines that reliably find relevant papers wherever they may be published. The era of AI will put this paper-journal dogma to the test like never before: much smaller units of “reproducible, fully documented” science will now become much easier to find, which, in principle, challenges the primacy of the research paper. Yet, for the foreseeable future, the paper—and only the paper—remains the unit of research assessment, underwriting its dominance in scientific dissemination.

If the paper is to remain the hub of knowledge, it has to be made fit for its purpose of sharing scientific findings. To achieve this, it needs to rediscover its heart and soul: the data. We need to dip beneath the figures to share research findings more optimally.

## Source data

All *EMBO Press* journals have encouraged source-data deposition for more than a dozen years (Lemberger, 2010; Pulverer, 2014). We have now closed the circle and require authors to submit the source data for all critical experiments in a paper. To support this policy, we have teamed up with the EBI (https://www.ebi.ac.uk) that developed a robust, free, and structured platform for data: BioStudies (https://www.ebi.ac.uk/biostudies/). Biostudy deposits provide citable source-data records and a long-term archiving service for all the key minimally processed data underlying a paper, including metadata, replicates, and all. EMBO data curators— increasingly supported by AI—assist in posting and rendering the experiments machine-readable by coding entities in a relational manner (Liechti et al, 2017; https://sourcedata.embo.org). We also apply the MDAR (Material Design Analysis Reporting) standard (Macleod et al, 2021)—now broadly adopted by quality journals—as well as the new PRO-MaP (Batista et al, 2023) recommendations for methods and protocols, to ensure posting in available structured repositories (https://www.embopress.org/page/journal/14693178/authorguide#datadeposition). We are aware of the extra effort involved, but pose that this is the only way to optimize reproducibility.

## Transparent to humans and machines

**Figure 1:**
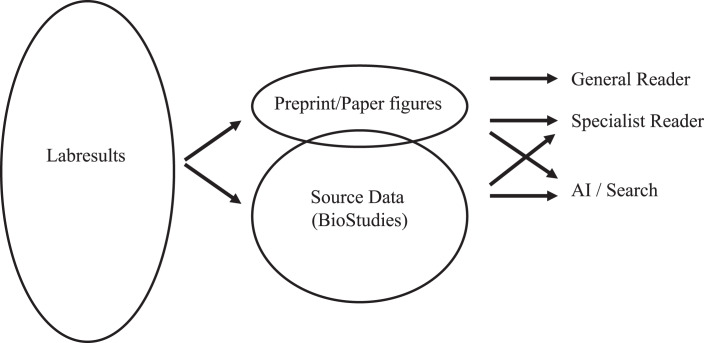
Data transparency in papers and preprints. Reproducible data underlying a research project are shared traditionally as figures optimized for visual inspection by the general reader and described in the figure legend and main text of the preprint or paper. Source data underlying research results in figures are also shared in 'minimally processed' form on structured community databases and now also in less structured form as source data on the Biostudies repository. This is crucial to open data to analysis and re-use by specialists and to computational analysis.

EMBO source data ticks off at least the first two letters of FAIR data sharing. In our view, this is a sustainable solution between pragmatism and idealism: the source data we provide—in structured data repositories where available or on Biostudies in a semi-structured form—allows browsing by human readers, but, crucially, also by AI (Fig. 1). One may speculate that the rapid advances in AI may induce a collective sigh of relief if it makes semi-structured data available for computational processing, even in the absence of standardized structured metadata.

## Microattribution: claim your experiments!

But there is more to source data than just being a machine-readable, long-term archive of minimally processed data. Papers are complex beasts that bundle many years of work, often across international collaborations. We capture authors’ contributions in the byline, in a broadly understood yet informal, largely hierarchical order from first to second, middle to—via a sidestep over acknowledgments—corresponding author. The increasing occurrence of quintuple first and/or corresponding authors may reflect more scientific cooperation, but it may also reflect the pressures to optimize one’s performance for research assessment. Requests for first-first authorship and co-second authorship—we allow neither—or the widespread perception that the last corresponding author is the “senior” author—this is only true in as far as they often pay the publication bill—support the notion that research assessment dominates authorship assignment. The adoption of CRediT (Contributor Roles Taxonomy; https://credit.niso.org) for a structured author contribution section - obligatory at this journal - has helped to add some level of transparency, standardization and nuance. However, as Howy Jacobs points out in this month’s issue, this is not enough (Jacobs, 2024). He argues that current authorship practices “only scratched the surface of proper attribution” and that “[authorship guidelines] do not adequately address the main difficulty, of deciding how to value each author’s work”.

Coming back to source data, we feel that we address Howy’s criticism by encouraging figure panel-level authorship. A paper’s source-data ‘avatar’ on BioStudies can now contain a tabular breakdown of who contributed what to every figure panel displayed. Although this facility is voluntary, almost 60% of our authors already contribute figure-level authorship. In due course, we hope that specific experiments can thus be credited more precisely to specific authors. This is particularly important to ensure that middle authors, who are currently largely invisible to research assessment, can document their contributions by associating their names with the experiments they performed.

In our view, figure panel authorship adds credit in a manner that is complementary to author contribution sections. We hope that as other journals adopt such policies, this more granular level of authorship can be rendered machine-readable and thus visible to research assessment. The often-painful disputes we witness in the jostle for “research-assessment relevant authorship positions” are testament to the urgent need for reform towards a more granular mapping of contributions.

## Credit and accountability

Claiming credit for specific experiments should help scientists to progress on the career ladder and secure funding, but it also adds transparency in terms of who did what. This additional level of accountability is pivotal in resolving complex data integrity issues: the journal and institutions know immediately who to ask for clarifications. Signing off on specific experiments will undoubtedly also lead to extra care in how the data are processed and presented in the figure. While we have no evidence for causality, the recent decrease in image integrity issue that we have observed may, at least partially, owe to authors publicly claiming credit for their experiments.

For figure panel/source-data level authorship to change the system, we need broader adoption. Ask your favorite journals to post source data and adopt figure panel level authorship. The literature has to move beyond telling stories about exciting science to underpin notable findings with the underlying data. The sweat and toil that went into countless replicates deserve to be seen.
